# Auxin-induced degradation of the aurora A kinase, AIR-1, in *C. elegans* does not prevent assembly of bipolar meiotic spindles

**DOI:** 10.17912/micropub.biology.001123

**Published:** 2024-01-31

**Authors:** Karen McNally, Francis McNally

**Affiliations:** 1 Molecular and Cellular Biology, University of California, Davis, Davis, California, United States

## Abstract

Chromosome segregation during mitosis and male meiosis is mediated by centrosomal spindles that require the activity of the aurora A kinase, whereas female meiotic spindles of many species are acentrosomal. We addressed the role of the *C. elegans* aurora A kinase, AIR-1 , in acentrosomal spindle assembly by generating a strain in which AIR-1 is tagged with both an auxin-induced degron and HALO tag. The meiotic spindle pole marker, MEI-1 , and chromosomes were labeled with GFP and mCH::histone respectively. All meiotic spindles were bipolar in AIR-1 depleted embryos, however an increase in lagging chromosomes was observed during anaphase.

**Figure 1. Depletion of AIR-1 results in bipolar meiotic spindles and an increased frequency of lagging chromosomes during anaphase:**
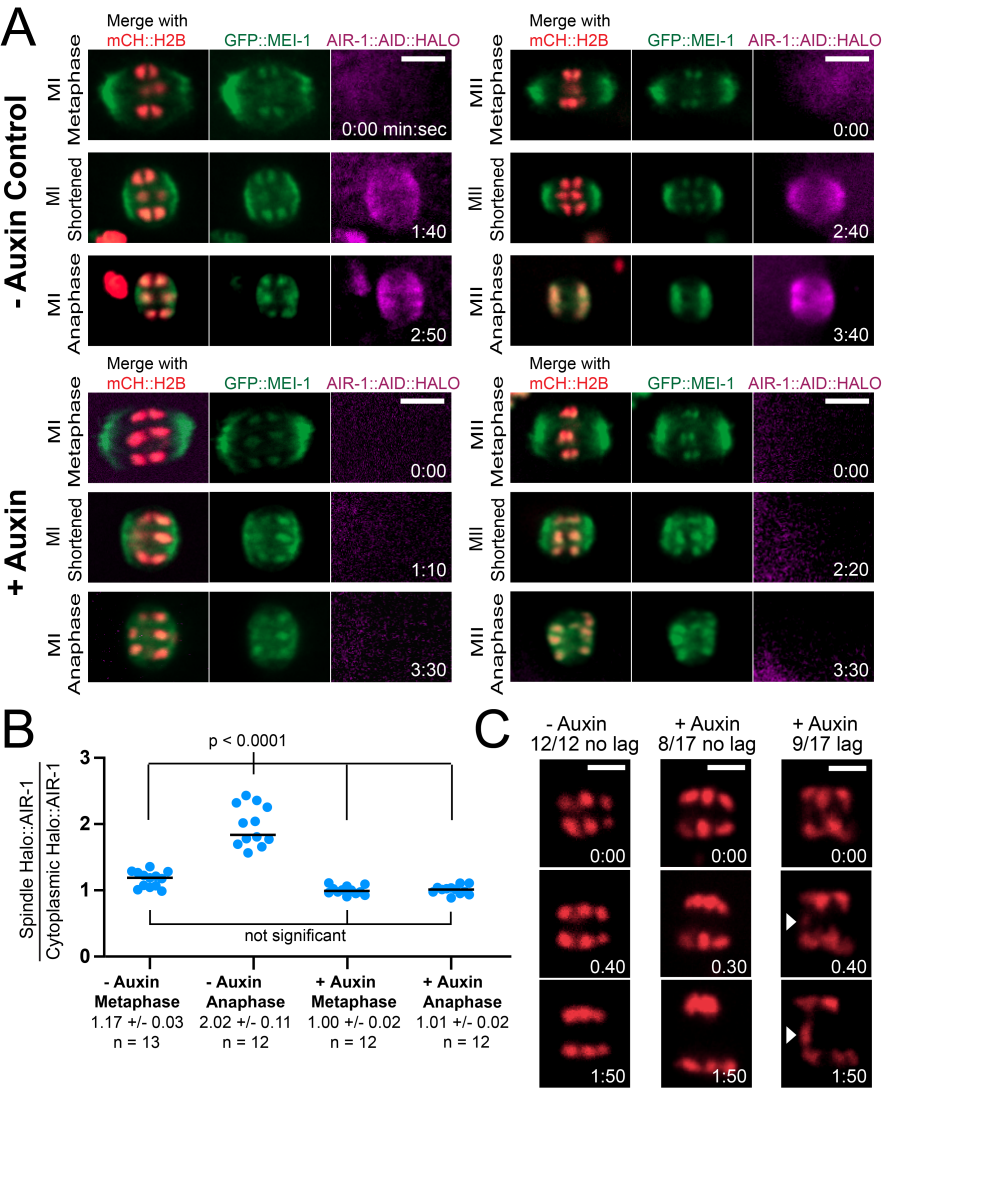
(A) Time-lapse images of female meiotic spindles were captured in Control and AIR-1 -depleted embryos expressing mCH::H2B, AIR-1::AID::HALO, and GFP:: MEI-1 , which localizes to spindle poles and chromosomes. Bipolar spindles were observed in 15/15 - Auxin Control embryos and in 20/20 + Auxin embryos. AIR-1::AID::HALO was observed on anaphase spindles in Control embryos and was depleted with overnight auxin treatment. All size bars = 4μm. (B) The ratio of spindle to cytoplasmic AIR-1::AID::HALO pixel intensity was determined for both metaphase and anaphase spindles in Control and + Auxin embryos. (C) Time-lapse images were captured in Control and AIR-1-depleted embryos expressing mCH::H2B. Arrowheads indicate the position of a lagging chromosome. All size bars = 3μm.

## Description

The *C. elegans* aurora A kinase homolog, AIR-1 , has been predominantly studied in one celled embryos where it acts as a centrosomal kinase and is required for recruitment of pericentriolar material (PCM) proteins and regulation of astral microtubules emanating from centrosomes (Schumacher et al., 1998; Hannak et al., 2001; Motegi et al., 2006; Davis et al., 2022) . It has also been found at the cortex of one celled mitotic embryos (Kotak et al., 2016; Klinkert et al., 2019) . C. elegans female meiotic spindles do not have centrioles (Albertson and Thomson, 1993) and do not have PCM proteins like g-tubulin at their poles (McNally et al., 2006) , therefore it was somewhat surprising when *air-1 (RNAi)* meiotic embryos were unable to form bipolar meiotic spindles and were unable to segregate meiotic chromosomes (Sumiyoshi et al., 2015) . *C. elegans* female meiotic spindle poles exhibit concentrations of ASPM-1 (Wignall and Villeneuve, 2009) , LIN-5 (van der Voet et al., 2009) , ZYG-9 (Chuang et al., 2020) , KLP-18 (Wolff et al., 2016) and MEI-1 (Clark-Maguire and Mains, 1994) at metaphase and specifically recruit cytoplasmic dynein and dynactin to their poles during anaphase (Ellefson and McNally, 2011) . The genetic requirements for meiotic spindle pole formation appear to be distinct from the requirements for centrosome formation. No meiotic spindle poles form in the absence of MEI-1 (McNally and McNally, 2011) , AIR-2 (Divekar et al., 2021) , cohesin (McNally et al., 2022) , or KLP-15 /16 (Mullen and Wignall, 2017) and meiotic spindle poles do not stably coalesce after depletion of KLP-18 (Wolff et al., 2016) , CLS-2 (Schlientz and Bowerman, 2020) , ZYG-9 (Cavin-Meza et al., 2022) , or KLP-7 (Connoly et al., 2015; Gigant et al., 2017).

To further investigate the role of AIR-1 in meiotic spindle function, we tagged the endogenous air-1 locus with an auxin-induced degron (AID) and fluorescent protein (HALO) in a strain with a germline expressed TIR1, an mCherry::histone H2b transgene, and the endogenous *mei-1* locus tagged with GFP.

Time-lapse in utero imaging of meiotic embryos within worms fed Janelia Fluor646 HaloTagLigand revealed that AIR-1 ::AID::HALO was very dimly associated with early metaphase spindles but became brighter at spindle poles during spindle shortening and anaphase A of both meiosis I and meiosis II ( Fig. 1A -B). Picking L4 larvae onto 4mM auxin plates resulted in 100% embryonic lethality (n=475 embryos, 3 worms) whereas the same strain without auxin yielded only 1.9% embryonic lethality (n=513 embryos, 3 worms). Auxin eliminated any discernable AIR-1 ::AID::HALO fluorescence ( Fig. 1A -B) on meiotic spindles. Auxin also eliminated any discernable centrosomal AIR-1 ::AID::HALO fluorescence in 20/20 mitotic embryos, whereas 15/18 mitotic embryos in control worms had bright centrosomal AIR-1 ::AID::HALO fluorescence. Localization of GFP:: MEI-1 to spindle poles and chromosomes as well as bipolar spindle assembly, spindle shortening and spindle rotation proceeded normally both with and without auxin ( Fig. 1A ). Roughly half of AIR-1 -depleted spindles exhibited a lagging chromosome during anaphase whereas no lagging chromosomes were observed among no auxin controls ( Fig. 1C ). Because we utilized single focal plane time-lapse imaging, it is possible that additional chromosomes out of the central focal plane lagged during anaphase or failed to congress at metaphase.

In theory, the stronger phenotypes published for air-1 (RNAi) and the drug MLN8237 (Sumiyoshi et al., 2015) might be due to off-target effects on the aurora B kinase, AIR-2 , which is required for bipolar meiotic spindle assembly and anaphase (Divekar et al., 2021) . However, Sumiyoshi et al. reported that an RNAi-resistant GFP:: AIR-1 transgene rescued the meiotic spindle phenotypes of 4/4 *air-1 (RNAi)* embryos. An alternate possibility is that our auxin-induced degradation left a higher amount of residual AIR-1 than the 2015 RNAi treatment.

## Methods

L4 larvae were transferred to 60mm plates seeded with OP50 and containing either 4mM auxin or ethanol solvent control. Janelia Flour®646 HaloTag® ligand (Promega Catalog #: GA1120) was diluted in M9 media to a final concentration of 2.5µM and 50µl was added directly to each plate. Worms were incubated for 24 hours in a dark 20°C incubator and then anesthetized by transferring adult hermaphrodites to a solution of 0.1% tricaine, 0.01% tetramisole in PBS for 30 min as described in Kirby et al. (1990) and McCarter et al. (1999). Worms were mounted between an agarose pad and coverslip as described in Danlasky et al. (2020) and subjected to time lapse imaging on a Yokogawa CSU-10 spinning disk confocal microscope equipped with an Olympus 100X 1.3 PlanApo objective and a Hammamatsu Orca Quest qCMOS detector. Exposures were captured every 10 seconds with sequential excitation by 488 nm, 647 nm and 561 nm lasers.

## Reagents

PHX7903 *mei-1 (or1937[GFP:: mei-1 ])I;cpIs103 [sun-1p::TIR1] II; itIs37 [pie-1p::mCherry:: his-58 ] IV; air-1 (syb7903 [ air-1 ::AID::HALO]) V*

available from fjmcnally@ucdavis.edu

## References

[R1] Albertson DG, Thomson JN (1993). Segregation of holocentric chromosomes at meiosis in the nematode, Caenorhabditis elegans.. Chromosome Res.

[R2] Cavin-Meza G, Mullen TJ, Czajkowski ER, Wolff ID, Divekar NS, Finkle JD, Wignall SM (2022). ZYG-9ch-TOG promotes the stability of acentrosomal poles via regulation of spindle microtubules in C. elegans oocyte meiosis.. PLoS Genet.

[R3] Chuang CH, Schlientz AJ, Yang J, Bowerman B (2020). Microtubule assembly and pole coalescence: early steps in Caenorhabditiselegans oocyte meiosis I spindle assembly.. Biol Open.

[R4] Clark-Maguire S, Mains PE (1994). Localization of the mei-1 gene product of Caenorhaditis elegans, a meiotic-specific spindle component.. J Cell Biol.

[R5] Connolly AA, Sugioka K, Chuang CH, Lowry JB, Bowerman B (2015). KLP-7 acts through the Ndc80 complex to limit pole number in C. elegans oocyte meiotic spindle assembly.. J Cell Biol.

[R6] Danlasky BM, Panzica MT, McNally KP, Vargas E, Bailey C, Li W, Gong T, Fishman ES, Jiang X, McNally FJ (2020). Evidence for anaphase pulling forces during C. elegans meiosis.. J Cell Biol.

[R7] Davis P, Zarowiecki M, Arnaboldi V, Becerra A, Cain S, Chan J, Chen WJ, Cho J, da Veiga Beltrame E, Diamantakis S, Gao S, Grigoriadis D, Grove CA, Harris TW, Kishore R, Le T, Lee RYN, Luypaert M, Müller HM, Nakamura C, Nuin P, Paulini M, Quinton-Tulloch M, Raciti D, Rodgers FH, Russell M, Schindelman G, Singh A, Stickland T, Van Auken K, Wang Q, Williams G, Wright AJ, Yook K, Berriman M, Howe KL, Schedl T, Stein L, Sternberg PW (2022). WormBase in 2022-data, processes, and tools for analyzing Caenorhabditis elegans.. Genetics.

[R8] Divekar NS, Davis-Roca AC, Zhang L, Dernburg AF, Wignall SM (2021). A degron-based strategy reveals new insights into Aurora B function in C. elegans.. PLoS Genet.

[R9] Ellefson ML, McNally FJ (2011). CDK-1 inhibits meiotic spindle shortening and dynein-dependent spindle rotation in C. elegans.. J Cell Biol.

[R10] Gigant E, Stefanutti M, Laband K, Gluszek-Kustusz A, Edwards F, Lacroix B, Maton G, Canman JC, Welburn JPI, Dumont J (2017). Inhibition of ectopic microtubule assembly by the kinesin-13 KLP-7 prevents chromosome segregation and cytokinesis defects in oocytes.. Development.

[R11] Hannak E, Kirkham M, Hyman AA, Oegema K (2001). Aurora-A kinase is required for centrosome maturation in Caenorhabditis elegans.. J Cell Biol.

[R12] Kirby C, Kusch M, Kemphues K (1990). Mutations in the par genes of Caenorhabditis elegans affect cytoplasmic reorganization during the first cell cycle.. Dev Biol.

[R13] Klinkert K, Levernier N, Gross P, Gentili C, von Tobel L, Pierron M, Busso C, Herrman S, Grill SW, Kruse K, Gönczy P (2019). Aurora A depletion reveals centrosome-independent polarization mechanism in Caenorhabditis elegans.. Elife.

[R14] Kotak S, Afshar K, Busso C, Gönczy P (2016). Aurora A kinase regulates proper spindle positioning in C. elegans and in human cells.. J Cell Sci.

[R15] McCarter J, Bartlett B, Dang T, Schedl T (1999). On the control of oocyte meiotic maturation and ovulation in Caenorhabditis elegans.. Dev Biol.

[R16] McNally K, Audhya A, Oegema K, McNally FJ (2006). Katanin controls mitotic and meiotic spindle length.. J Cell Biol.

[R17] McNally KP, Beath EA, Danlasky BM, Barroso C, Gong T, Li W, Martinez-Perez E, McNally FJ (2022). Cohesin is required for meiotic spindle assembly independent of its role in cohesion in C. elegans.. PLoS Genet.

[R18] McNally KP, McNally FJ (2011). The spindle assembly function of Caenorhabditis elegans katanin does not require microtubule-severing activity.. Mol Biol Cell.

[R19] Motegi F, Velarde NV, Piano F, Sugimoto A (2006). Two phases of astral microtubule activity during cytokinesis in C. elegans embryos.. Dev Cell.

[R20] Mullen TJ, Wignall SM (2017). Interplay between microtubule bundling and sorting factors ensures acentriolar spindle stability during C. elegans oocyte meiosis.. PLoS Genet.

[R21] Schlientz AJ, Bowerman B (2020). C. elegans CLASP/CLS-2 negatively regulates membrane ingression throughout the oocyte cortex and is required for polar body extrusion.. PLoS Genet.

[R22] Schumacher JM, Ashcroft N, Donovan PJ, Golden A (1998). A highly conserved centrosomal kinase, AIR-1, is required for accurate cell cycle progression and segregation of developmental factors in Caenorhabditis elegans embryos.. Development.

[R23] Sumiyoshi E, Fukata Y, Namai S, Sugimoto A (2015). Caenorhabditis elegans Aurora A kinase is required for the formation of spindle microtubules in female meiosis.. Mol Biol Cell.

[R24] van der Voet M, Berends CW, Perreault A, Nguyen-Ngoc T, Gönczy P, Vidal M, Boxem M, van den Heuvel S (2009). NuMA-related LIN-5, ASPM-1, calmodulin and dynein promote meiotic spindle rotation independently of cortical LIN-5/GPR/Galpha.. Nat Cell Biol.

[R25] Wignall SM, Villeneuve AM (2009). Lateral microtubule bundles promote chromosome alignment during acentrosomal oocyte meiosis.. Nat Cell Biol.

[R26] Wolff ID, Tran MV, Mullen TJ, Villeneuve AM, Wignall SM (2016). Assembly of Caenorhabditis elegans acentrosomal spindles occurs without evident microtubule-organizing centers and requires microtubule sorting by KLP-18/kinesin-12 and MESP-1.. Mol Biol Cell.

